# 'No collaboration, no trial; why collaborator opinion matters'

**DOI:** 10.1186/1745-6215-14-S1-P127

**Published:** 2013-11-29

**Authors:** Claire Cochran

**Affiliations:** 1 University of Aberdeen, Aberdeen, UK

## 

For a trial to be successful, site personnel must retain high commitment to a trial often over long periods of time. Little evidence exists, however, to guide what type of communication/training activities should be adopted to help promote long-term collaboration. In this presentation, we report the role of focus groups to inform the appropriate choice of communication/training activities for a large international study.

EAGLE (**E**ffectiveness, in **A**ngle closure **G**laucoma, of **L**ens **E**xtraction) is an international multi-centre, pragmatic, randomised controlled trial (RCT). Within EAGLE trial data from participant questionnaires and clinical case report forms are uploaded onto the bespoke EAGLE website by collaborators at 31 sites worldwide. In order to elicit useful experiences and opinions from collaborators to inform future trial procedures, focus groups were conducted during a trial training event.

Group training days, rather than individual visits, were preferred as they provide an opportunity to network with peers and focus on the trial away from distraction of the everyday environment. Collaborator newsletters remain a popular way of maintaining trial profile at a group level; at individual level distributing tasks by regular brief emails encourages engagement. Web based data entry gives collaborators a sense of ownership over the data they collect, but thoughtful website design and (telephone) support is key to maintaining data quality. Collaborators like trials that mimic standard clinical care; they are perceived easier to recruit and retain patients than other explanatory trials. Barriers included complexity of the inclusion and exclusion criteria, and issues with local financial support.

